# Effect of Prehospital Epinephrine on Outcomes of Out-of-Hospital Cardiac Arrest: A Bayesian Network Approach

**DOI:** 10.1155/2020/8057106

**Published:** 2020-08-01

**Authors:** Joonghee Kim, Yu Jin Kim, Sangsoo Han, Han Joo Choi, Hyungjun Moon, Giwoon Kim

**Affiliations:** ^1^Department of Emergency Medicine, Seoul National University Bundang Hospital, 82, Gumi-ro 173 Beon-gil Bundang-gu, Seongnam-si Gyeonggi-do, Seongnam 13620, Republic of Korea; ^2^Department of Emergency Medicine, Soonchunhyang University Bucheon Hospital, 170, Jomaru-ro Bucheon-si Gyeonggi-do, Bucheon 14584, Republic of Korea; ^3^Department of Emergency Medicine, Dankook University Hospital, 201, Manghyang-ro Dongnam-gu, Cheonan-si Chungcheongnam-do, Cheonan 31116, Republic of Korea; ^4^Department of Emergency Medicine, Soonchunhyang University Hospital, 44, Suncheonhyang 4-gil Dongnam-gu, Cheonan-si Chungcheongnam-do, Asan 31151, Republic of Korea

## Abstract

**Background:**

The benefit of prehospital epinephrine in out-of-hospital cardiac arrest (OHCA) was shown in a recent large placebo-controlled trial. However, placebo-controlled studies cannot identify the nonpharmacologic influences on concurrent or downstream events that might modify the main effect positively or negatively. We sought to identify the real-world effect of epinephrine from a clinical registry using Bayesian network with time-sequence constraints.

**Methods:**

We analyzed a prospective regional registry of OHCA where a prehospital advanced life support (ALS) protocol named “Smart ALS (SALS)” was gradually implemented from July 2015 to December 2016. Using Bayesian network, a causal structure was estimated. The effect of epinephrine and SALS program was modelled based on the structure using extended Cox-regression and logistic regression, respectively.

**Results:**

Among 4324 patients, SALS was applied to 2351 (54.4%) and epinephrine was administered in 1644 (38.0%). Epinephrine was associated with faster ROSC rate in nonshockable rhythm (HR: 2.02, 6.94, and 7.43; 95% CI: 1.08–3.78, 4.15–11.61, and 2.92–18.91, respectively, for 1–10, 11–20, and >20 minutes) while it was associated with slower rate up to 20 minutes in shockable rhythm (HR: 0.40, 0.50, and 2.20; 95% CI: 0.21–0.76, 0.32–0.77, and 0.76–6.33). SALS was associated with increased prehospital ROSC and neurologic recovery in noncardiac etiology (HR: 5.36 and 2.05; 95% CI: 3.48–8.24 and 1.40–3.01, respectively, for nonshockable and shockable rhythm).

**Conclusions:**

Epinephrine was associated with faster ROSC rate in nonshockable rhythm but slower rate in shockable rhythm up to 20 minutes. SALS was associated with improved prehospital ROSC and neurologic recovery in noncardiac etiology.

## 1. Introduction

Current guidelines recommend epinephrine for advanced life support (ALS) in out-of-hospital cardiac arrest (OHCA) [1, 2]. After a long debate about its efficacy in OHCA, Perkins et al. proved the drug can improve the chance of survival in a large randomized placebo-controlled double-blind study (RCT) [3, 4]. The study objectively assessed the averaged pharmacologic effect of the drug in OHCA. However, if we see the aspect of epinephrine use as an ALS procedure, rather than just focusing on the pharmacologic effect, it is still not clear how its use will manifest in real-world situation because the procedural aspect, such as securing an intravenous access which was controlled in the study by using saline placebo, can have significant impact on other concurrent or downstream events and procedures that might significantly modify the main effect of epinephrine in a positive or a negative way.

One alternative approach that can access the real-world effect of epinephrine as an ALS procedure will be conducting an observational study using clinical registries. However, the treatment allocation in real-world situation is never random and thus conditioning the appropriate variables is very important. Specifically, one need to control a set of confounders that will close every open pathway between exposure and outcome variables while being cautious not to open a closed pathway by conditioning colliders [5, 6]. Therefore, it is required to know a priori the web of influences between significant factors before main analysis.

Defining the causal structure in OHCA is difficult because its outcomes are determined by complex interplays of multiple factors. It is a common practice that an expert build it based on his/her knowledge and experience for further causal analysis. However, either the lack of previous study or the presence of multiple previous studies with conflicting results as well as any biased personal opinions of the expert might lead to suboptimal causal structure. In addition, it should be considered that the causal relationships can be heterogeneous by study design and setting [7, 8].

Therefore, in present study, we take a data-driven approach to secure the causal structure and assess the effect of epinephrine in prehospital resuscitation. Specifically, we use Bayesian network, a computational methodology for analysis of the conditional dependencies between variables, to identify causal pathways in OHCA [9]. Then, we model the effect of epinephrine on outcomes utilizing the structure of the causal pathways. Therefore, the objective of the present study is twofold. The first one is to identify the causal structure in OHCA. The second one is to estimate the effect of prehospital epinephrine based on the structure.

## 2. Methods

### 2.1. Study Setting, Participants, and Data Source

A prehospital ALS protocol named “Smart Advanced Life Support (SALS)” was gradually implemented in 7 urban and suburban areas of South Korea from July 2015 to December 2016. If SALS is applied, ALS procedures are provided by emergency medical technicians (EMTs) under direct medical control. The procedures include advanced airway and repeated IV epinephrine bolus at least for 20 minutes on scene if there is no ROSC. A dedicated smart phone-based video call system was used for the medical control. The protocol requires a second ambulance available for help in the same area and an informed consent by the caregiver. If any of the two is not available, BLS is provided instead of ALS without any use of epinephrine for at least 5 minutes on scene. The implementation of prehospital ALS was the first attempt in Korea.

The data source was a regional OHCA registry initiated for quality assurance of SALS. It includes various Utstein- and SALS-related information and is managed by dedicated researchers allocated to each participating area. OHCA cases excluded from registry as well as the present study were irreversible death, age less than 18, do-not-resuscitate status, and traumatic cardiac arrest. Cardiac arrest monitored by EMTs was additionally excluded. The institutional review board at our hospital approved the analysis and waived the requirement of informed consent (IRB Number: AJIRB-TEMP-TEMP-15-516).

### 2.2. Statistical Analysis

A Bayesian network is a probabilistic graphical model that represents a set of variables and their conditional dependencies via a directed acyclic graph (DAG) [9–12]. Each vertex (variable) has a probability function that takes a set of values from its parents and gives the probability distribution of the state of the vertex. We used discrete Bayesian network where all vertices are represented by finite and discrete states [11].

We used the following variables: sex, age, presumed etiology, public location, witnessed cardiac arrest, response time, bystander CPR, initial rhythm, application of SALS, epinephrine use, advanced airway, time to emergency department (ED) arrival (EDA time), prehospital ROSC, and neurologic recovery (6-month cerebral performance category [CPC] score 1 or 2). Because we are using discrete Bayesian network, we categorized continuous variables to quartiles: age: <59, 59–72, 73–81 and >81, response time: <7, 7,8–10 and >10, EDA time: <19, 19–26, 27–35 and >35.

Generating a Bayesian network is a two-step process. We first construct its structure and then estimate its parameters. We determined a priori the hierarchy of possible influences among variables by imposing chronological orders (Supplemental Table 1, Supplemental Figure 1). This inhibits pathways that are absolutely impossible (e.g., influence to preceding events or attributes) being generated during structural estimation. Its first tier includes sex and age and the second one is presumed etiology. The third one includes location-related factors such as public location, witnessed arrest, and response time. The fourth tier is bystander CPR and the fifth tier is shockable initial rhythm. The sixth tier includes SALS, ALS procedures (epinephrine and advanced airway), EDA time, and prehospital ROSC. In this tier, there are additional constraints that allow only the influences from SALS to each ALS procedure but not in reverse direction. The last tier is neurologic recovery. The structural learning is briefly summarized as follows [11, 12]. We generate 2,000 datasets with the same number of cases as the original dataset using bootstrapped resampling. Then, we apply hill climbing algorithm to generate an optimal network structure for each resampled dataset. The algorithm starts from a DAG with no edge, then it adds, removes and reverses one edge at a time, and picks the change that increases the network score the most. The network score used was Bayesian Dirichlet equivalent uniform score. It is a posterior probability of a candidate graph with a flat Dirichlet prior over both the space of DAGs and the parameter space of each node. It takes the form of

(1) $$\begin{array}{c} \left\{B\;De\;u\left(G,D\right)=\overset{p}{\underset{i=1}{\prod }}\overset{q_{i}}{\underset{j=1}{\prod }}\left\{\frac{\Gamma \left(\alpha_{ij}\right)}{\Gamma \left(\alpha_{ij}+n_{ij}\right)}\overset{r_{i}}{\underset{k=1}{\prod }}\frac{\Gamma \left(\alpha_{ijk}+n_{ijk}\right)}{\Gamma \left(\alpha_{ijk}\right)}\right\}\right\}, \end{array}$$

where *G* is a DAG, *D* is a dataset, *α* is the imaginary sample size associated with the Dirichlet prior, *p* is the number of nodes in *G*, *q*_*i*_ is the number of categories for the node *X*_*i*_, *r*_*i*_ is the number of configurations of the categories of the parents of *X*_*i*_, and *n*_*ijk*_ is the number of samples for the *j*th category for node *X*_*i*_ and *k*th configuration for its parents. Then model averaging is performed by determining whether the probability of each possible edge present in the true network structure is larger than a threshold. The threshold was determined as the value minimizing the *L*1-norm between the cumulative distribution function of the observed edge confidences and those of its asymptotic counterpart [13]. After structural learning, model parameters are estimated based on Bayesian method using non-informative prior.

We assessed the effect of epinephrine in two ways. The first one is to estimate the effect of a treatment (e.g., epinephrine) on ROSC rate during an ongoing resuscitation. The second one is to estimate the effect of a treatment decision (e.g., SALS) on prehospital ROSC and neurologic recovery, whose rationale will be discussed later. The effect on ROSC rate was modelled by constructing an extended Cox-regression model where prehospital epinephrine is included as a time varying covariate [14]. In this model, the resuscitation duration was split into three segments (*t* < 10 minutes, 10 ≤ *t* < 20 minutes, *t* ≥ 20 minutes) and the effect size of epinephrine was assumed to be constant within each segment. The effect of SALS on prehospital ROSC and neurologic recovery was modelled using logistic regression. In both of the models, covariates were chosen based on the DAG and interactions were checked up to first order. Lastly, we repeated the same analysis by querying the Bayesian network itself.

All variables were reported using frequency and proportion. Chi-square or Fisher's exact test was used to compare the groups. *P* values <0.05 were considered significant. All statistical analyses were performed using the R package version 3.3.0 (R Foundation for Statistical Computing, Vienna, Austria).

## 3. Results

A total of 4745 OHCA victims were identified. Excluding monitored cardiac arrest (*N* = 417, 8.8%) and incomplete data entry (*N* = 4, 0.1%), a total of 4,324 patients (91.1%) were included. SALS was applied to 2351 (54.4%) patients (Table 1). SALS was associated with younger median age (53 vs. 56 years; IQR, 38–62 vs. 41–64; *P* < 0.001), presumed cardiac etiology (17.2% vs. 3.4%; *P* < 0.001), bystander CPR (67.8% vs. 60.0%; *P* < 0.001), and shockable initial rhythm (19.7% vs. 13.8%; *P* < 0.001). Epinephrine was administered only in SALS group (69.9% vs. 0.0%; *P* < 0.001) while advanced airway was applied in both groups with higher frequency in SALS group (95.9% vs. 53.7%; *P* < 0.001). SALS group required significantly more time until ED arrival (28 vs 14 minutes; IQR, 22–35 vs. 10–20; *P* < 0.001); however, it showed higher prehospital ROSC (13.8% vs. 4.2%; *P* < 0.001) and neurologic recovery (6.3% vs. 2.6%; *P* < 0.001).

**Figure 1:**
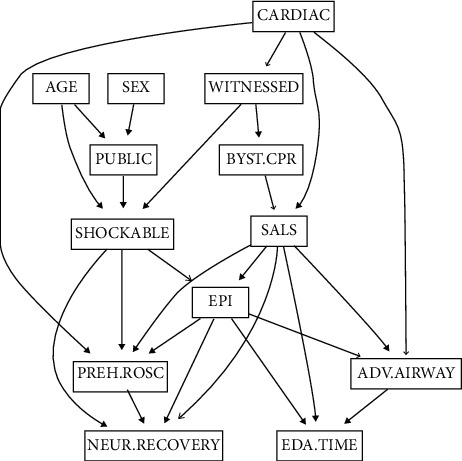
The directed acyclic graph (DAG) structure of the Bayesian network constructed using bootstrap model averaging. Arrow indicates the direction of influence and line thickness corresponds to the relative edge strength. Smart advance life support (SALS) was dependent on presumed etiology and bystander cardiopulmonary resuscitation (CPR) while epinephrine was dependent on SALS and initial rhythm. Prehospital return of spontaneous circulation (ROSC) was dependent on initial rhythm, SALS, epinephrine, and presumed etiology. Neurologic recovery was dependent on prehospital ROSC, initial rhythm, epinephrine, and SALS. Advanced airway was dependent on SALS, epinephrine, and presumed etiology; however, it did not have any direct or indirect pathway leading to outcome variables. CARDIAC, presumed cardiac etiology; WITNESSED, witnessed cardiac arrest; PUBLIC, public location; BYST.CPR, bystander cardiopulmonary resuscitation; SALS, Smart advance life support; EPI, epinephrine use; PREH.ROSC, prehospital return of spontaneous circulation; ADV.AIRWAY, advanced airway; NEUR.RECOVERY, neurologic recovery; EDA.TIME, emergency department arrival time.

A DAG was constructed using bootstrap model averaging (Figure 1). SALS was dependent on presumed etiology and bystander CPR while epinephrine was dependent on SALS and initial rhythm. Prehospital ROSC was dependent on initial rhythm, SALS, epinephrine, and presumed etiology. Neurologic recovery was dependent on prehospital ROSC, initial rhythm, epinephrine, and SALS. Advanced airway was dependent on SALS, epinephrine, and presumed etiology; however, it did not have any direct or indirect pathway leading to outcome variables. Based on the structure, model parameters were estimated using Bayesian method with non-informative prior. Conditional probabilities of exposure and outcome vertices (SALS, epinephrine, prehospital ROSC, and neurologic recovery) are summarized in supplemental Figure 2.

**Figure 2:**
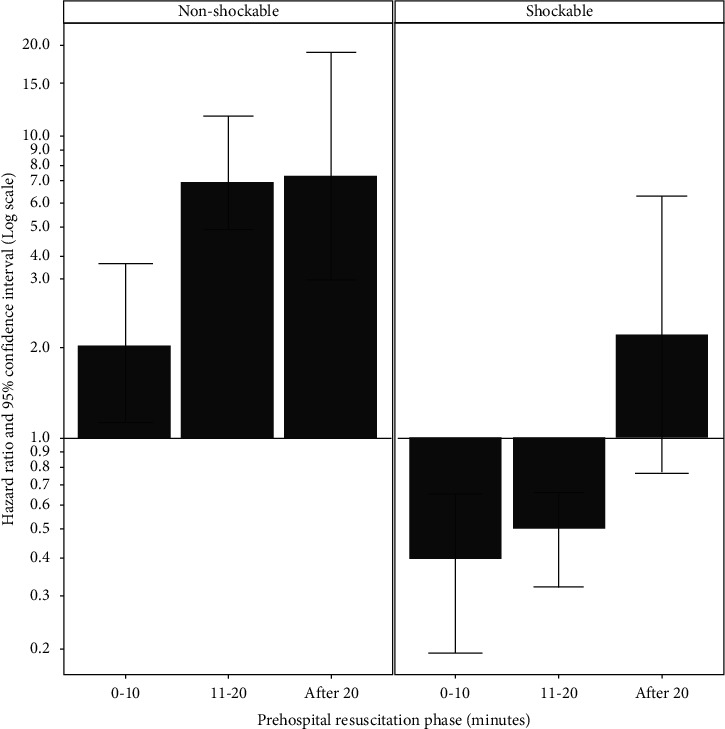
Effect of epinephrine on prehospital ROSC rate. Epinephrine use was associated with faster ROSC rate in nonshockable initial rhythm (left), while it was associated with slower ROSC rate up to 20 minutes in shockable initial rhythm (right).

The effect of epinephrine on ROSC rate was estimated using extended Cox-regression (Table 2). Shockable initial rhythm and SALS were included as covariates based on the DAG. With a significant negative interaction between epinephrine and shockable initial rhythm, we estimated the effect of epinephrine in shockable and nonshockable rhythm using linear combination (Figure 2). While epinephrine was associated with faster ROSC in nonshockable initial rhythm with HR of 2.02 (95% CI, 1.08–3.78; *P*=0.028), 6.94 (95% CI, 4.15–11.61; *P* < 0.001), and 7.43 (95% CI, 2.92–18.91; *P* < 0.001), respectively, for each resuscitation phase, it was associated with slower rate up to 20 minutes in shockable initial rhythm with HR of 0.40 (95% CI, 0.21–0.76; *P*=0.005), 0.50 (95% CI, 0.32–0.77; *P*=0.002), and 2.20 (95% CI, 0.76–6.33; *P*=0.146; supplemental Table 2), respectively.

**Figure 3:**
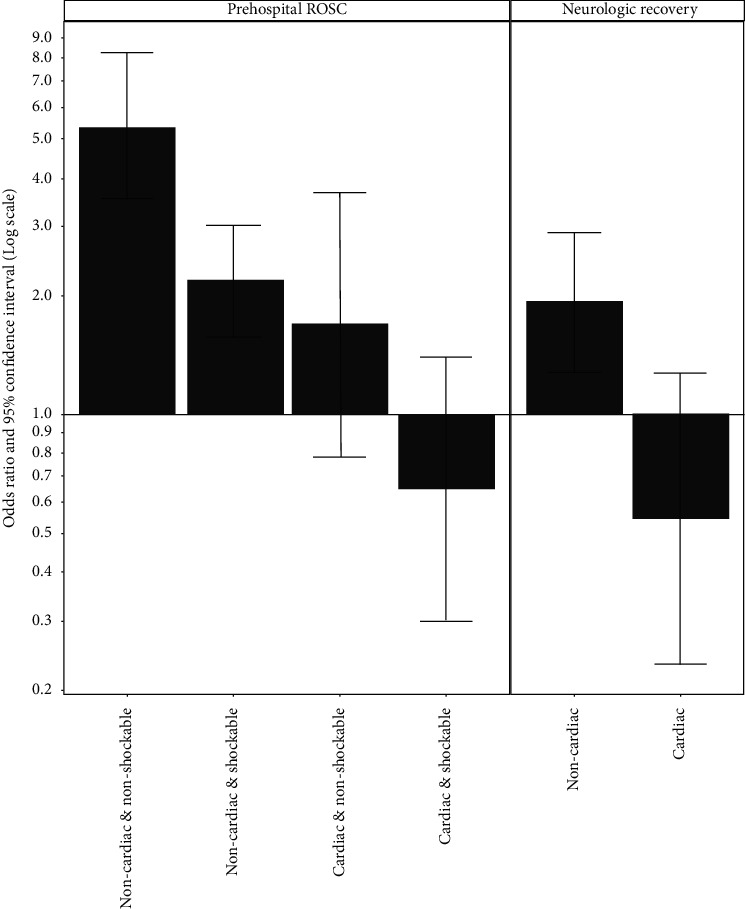
Effect of SALS intervention on prehospital ROSC and neurologic recovery. SALS was beneficial only in noncardiac etiology for both of the outcomes.

We assessed the effect of SALS on prehospital ROSC and neurologic recovery using logistic regression (supplemental Table 3). Covariates were selected based on the same DAG structure. SALS was associated with increased prehospital ROSC with OR of 5.36 (95% CI, 3.54–8.41, *P* < 0.001). However, SALS showed significant negative interaction with both shockable initial rhythm and presumed cardiac etiology (*P*=0.001 and *P*=0.004, respectively). SALS was associated with increased neurologic recovery with OR of 1.94 (95% CI, 1.31–2.91; *P*=0.001). However, there was significant negative interaction between SALS and presumed cardiac etiology (*P*=0.008).  Figure 3 summarizes the effect of SALS in each combinatorial states of the covariates with significant interaction. SALS was beneficial only in noncardiac etiology for both of the outcomes. The HRs for prehospital ROSC were 5.36 (95% CI, 3.48–8.24; *P* < 0.001) and 2.05 (95% CI, 1.40–3.01; *P* < 0.001) for nonshockable and shockable initial rhythm, respectively. The HR for neurologic recovery was 1.94 (95% CI, 1.30–2.89; *P*=0.001; supplemental Table 4). Direct querying of the Bayesian network showed similar results (Supplemental Figure 3).

## 4. Discussion

In this study, we mapped the network of influences in OHCA using Bayesian network. The network, which was given in the form of DAG, was used as a guidance to model the effect of epinephrine on prehospital ROSC and neurologic recovery. We found the effect of epinephrine is dependent on the context. It was associated with faster ROSC rate if initial rhythm was nonshockable, but slower rate up to 20 minutes if initial rhythm was shockable. In addition, SALS was beneficial only if presumed etiology was noncardiac. These suggest prehospital resuscitation strategy should be differentiated by initial rhythm, resuscitation phase, and presumed etiology.

Bayesian network can help to identify causal pathways [9–12] and has been used in clinical studies [15–17]. However, statistical relationships alone are not sufficient for finding a unique causal graph. It can only narrow down possible graphs that are Markov equivalent. Therefore, we applied chronological hierarchy to the structural learning, because temporal information can provide additional cues to a causal structure [10, 18]. However, temporal information can only be used to rule out improbable pathways and does not provide sufficient cue to rule them in. Despite the limitation, this data-driven approach has several merits over the traditional expert-driven approach. First, building a DAG structure by integrating expert's fragments of causal knowledge is difficult and prone to be biased by personal beliefs. Second, there could be either the lack of previous study or the presence of multiple previous studies with conflicting results in each candidate causal pathway. Third, some known causal relationships could be just association. Fourth, the causal relationships can be heterogeneous by study design or setting [7, 8].

There were two previous RCTs testing the effect of epinephrine in OHCA before the recent one by Perkins et al. Olasveengen et al. compared IV placement plus epinephrine (*N* = 418) with control (*N* = 433) and discovered the intervention improves short-term outcomes in nonshockable initial rhythm [19]. Jacobs et al. compared epinephrine (*N* = 272) with placebo (*N* = 262), in which epinephrine improved short-term outcomes. However, both of the studies were not powered enough to detect long-term benefits [20]. In the recent RCT by Perkins et al., a total of 8,014 patients were assigned to receive either epinephrine or saline placebo administered by an intravenous or intraosseous route every 3 to 5 minutes [4]. The use of epinephrine was associated with increased primary outcome (30-day survival, 3.2% vs. 2.4%) and long-term survival (3-month survival, 3.0% vs. 2.2%). In the subgroup analysis for the primary outcome, there was no statistically significant interaction. However, the favorable effect of the epinephrine was statistically significant only when the arrest was unwitnessed or the initial rhythm was nonshockable. The neurologic recovery measured at three months was 2.1% in the epinephrine arm and 1.6% in the placebo arm. However, the study was not powered enough to assess the significance of the difference.

There were several large-scale observational studies. Hagihara et al. analyzed Japan national OHCA registry (2005–2008) using propensity matching and reported prehospital epinephrine was associated with worse 1-month survival and functional outcome [21]. Nakahara et al. analyzed the same registry (2007–2010) using different statistical method (time-dependent propensity score matching) and reported the opposite results; prehospital epinephrine improved overall survival with small but statistically significant functional gain in nonshockable initial rhythm [22]. Fukuda et al. analyzed the same registry (2011–2012) using standard propensity matching and reported decreased 1-month survival and neurologic outcome with epinephrine use [23]. In most of these studies including the three RCTs previously mentioned, the effect of epinephrine was more favorable in nonshockable rhythm. The effect was stronger if it was beneficial while the effect was milder if was detrimental. This dependency on context was also observed in our study. There was strong interaction between epinephrine use and its contexts including initial rhythm, resuscitation phase, and presumed etiology. Unlike previous studies, we statistically demonstrated the presence of these interactions in our models.

The slower ROSC rate with epinephrine use in shockable initial rhythm needs further discussion. Conventionally, effect of an intervention on ROSC has been assessed by comparing the proportion of the outcome variables. However, this leads to biased estimate of its effect because doing some intervention increases the duration of prehospital phase which will in turn increase the chance of having prehospital ROSC. Therefore, we adopted extended Cox-regression to see if the intervention actually increases the speed of ROSC [24]. The mechanism for the slower ROSC rate up to 20 minutes is not clear. It is possible that additional efforts for epinephrine administration could have detrimental effect on overall quality of CPR. In the paper by Olasveengen et al., the quality of resuscitation was not affected by IV and epinephrine use [19]. However, it was an RCT involving highly trained EMTs and field anesthesiologists. In real world, administration of epinephrine can have significant negative impact on overall quality of resuscitation.

We used SALS intervention (intention-to-treat approach) instead of epinephrine use (per-treatment approach) as an exposure variable to assess the effect of epinephrine on neurologic recovery. It was because the time of ROSC influences both exposure (epinephrine) and outcomes (ROSC and neurologic recovery) and can induce significant biases without proper control [25]. This problem was recognized by Nakahara et al. and the authors used time-dependent propensity score matching to control the bias [22]. We could have adopted the same approach; however, we were not sure whether it can estimate the effect size as a whole. It matches a patient with first epinephrine administration at certain time point with those without it but has the same chance at that time. Therefore, what the method really assesses is the difference of outcomes between patients with earlier and later (or no) administration. Therefore, we used treatment decision (SALS) which is made at the beginning of resuscitation and thus not dependent on the duration of CPR. We admit the other component of SALS, advanced airway, could also have influenced the outcomes. However, we think the influence should be small because there was no direct or indirect causal pathway between advanced airway and any outcome in the DAG structure we identified.

## 5. Limitations

Our study has several limitations. Firstly, it is a retrospective observational study, and biases intrinsic to the design could have influenced the results. Secondly, the quality of prehospital resuscitation, which can have significant confounding effects, was not considered because of the absence of related data. Thirdly, the DAG structure we identified using Bayesian network is a statistical estimation of causal pathways. Although we disallowed absolutely impossible causal pathways only, it is possible the resulting DAG structure is not sufficiently comprehensive.

## 6. Conclusions

Prehospital use of epinephrine was associated with faster ROSC rate if initial rhythm was nonshockable, but slower rate up to 20 minutes if initial rhythm was shockable. Prehospital ALS protocol mandating epinephrine administration was associated with improved prehospital ROSC and neurologic outcome if presumed etiology was noncardiac.

## Figures and Tables

**Table 1:** Patient characteristics of the study population.

Characteristics	SALS applied (*N* = 2351)	Not applied (*N* = 1973)	*P*
Sex, male (%)	1593 (67.8%)	1197 (60.7%)	<0.001
Age, years, median (IQR)	53.0 (38.0–62.0)	56.0 (41.0–64.0)	<0.001
1^st^ quartile: <59 years	671 (28.5%)	474 (24.0%)	
2^nd^ quartile: 59–72 years	612 (26.0%)	449 (22.8%)	
3^rd^ quartile: 73–81 years	626 (26.6%)	517 (26.2%)	
4^th^ quartile: >81 years	442 (18.8%)	533 (27.0%)	
Presumed etiology, cardiac (%)	404 (17.2%)	67 (3.4%)	<0.001
Public location (%)	354 (15.1%)	296 (15.0%)	0.994
Witnessed cardiac arrest (%)	1131 (48.1%)	900 (45.6%)	0.109
Bystander CPR (%)	1595 (67.8%)	1184 (60.0%)	<0.001
Shockable initial rhythm (%)	464 (19.7%)	273 (13.8%)	<0.001
Response time, minutes, median (IQR)	7 (6–9)	7 (6–10)	0.195
1^st^ quartile: <7 minutes	868 (36.9%)	762 (38.6%)	
2^nd^ quartile: 7 minutes	399 (17.0%)	243 (12.3%)	
3^rd^ quartile: 8–10 minutes	681 (29.0%)	544 (27.6%)	
4^th^ quartile: >10 minutes	403 (17.1%)	424 (21.5%)	
Epinephrine (%)	1644 (69.9%)	0 (0.0%)	<0.001
Advanced airway (%)	2254 (95.9%)	1060 (53.7%)	<0.001
EMS time, minutes, median (IQR)	28 (22–35)	14 (10–20)	<0.001
1^st^ quartile: <19 minutes	143 (6.1%)	988 (50.1%)	
2^nd^ quartile: 19–26 minutes	498 (21.2%)	641 (32.5%)	
3^rd^ quartile: 27–35 minutes	834 (35.5%)	237 (12.0%)	
4^th^ quartile: >35 minutes	876 (37.3%)	107 (5.4%)	
Prehospital ROSC (%)	324 (13.8%)	83 (4.2%)	<0.001
Early ROSC (≤8 minutes)	97 (4.1%)	116 (5.9%)	0.010
Neurologic recovery (6-month CPC score 1 or 2)	148 (6.3%)	52 (2.6%)	<0.001

SALS, smart ALS (protocol); IQR, interquartile range; CPR, cardiopulmonary resuscitation; EMS, emergency medical service; ROSC, return of spontaneous circulation; CPC, cerebral performance category.

**Table 2:** Extended Cox-regression model for the effect of epinephrine on prehospital ROSC rate.

Predictor	Phase 1 (0–10 minutes)		Phase 2 (11–20 minutes)		Phase 3 (>20 minutes)	
	HR (95% CI)	*P*	HR (95% CI)	*P*	HR (95% CI)	*P*
SALS	0.56 (0.43–0.72)	<0.001	2.15 (1.36–3.39)	0.001	1.04 (0.36–3.01)	0.936
Shockable	19.42 (14.43–26.12)	<0.001	21.71 (13.05–36.12)	<0.001	6.18 (2.14–17.83)	0.001
Epinephrine: SALS	2.02 (1.08–3.78)	0.028	6.94 (4.15–11.61)	<0.001	7.43 (2.92–18.91)	<0.001
Epinephrine: SALS : shockable	0.20 (0.08–0.47)	<0.001	0.07 (0.04–0.13)	<0.001	0.3 (0.1–0.91)	0.033

ROSC, return of spontaneous circulation; HR, hazard ratio; SALS, smart ALS.

## Data Availability

The data used to support the findings of this study are available from the corresponding author upon request.

## Supplementary Materials

Supplementary Materials Supplemental Figure 1: potential causal pathways allowed to be included in the DAG structure. Supplemental Figure 2: conditional probabilities of exposure and outcome vertices. Supplemental Figure 3: effect of Epinephrine and SALS intervention assessed by network querying. Supplemental Table 1: definition of terms and detailed criteria used in this study. Supplemental Table 2: effect of epinephrine on prehospital ROSC rate. Supplemental Table 3: multivariable logistic regression model for the effect of SALS protocol on prehospital ROSC and neurologic recovery (6-month CPC score 1 or 2). Supplemental Table 4: effect of SALS intervention on prehospital ROSC and neurologic recovery.
