# Supplementary material for: Effect of Prehospital Epinephrine on Outcomes of Out-of-Hospital Cardiac Arrest: A Bayesian Network Approach
Source: Emerg Med Int. 2020 Aug 1;2020:8057106. doi: 10.1155/2020/8057106 (PMC7416253; doi:10.1155/2020/8057106)
Supplement: Supplementary Materials — Supplemental Figure 1: potential causal pathways allowed to be included in the DAG structure. Supplemental Figure 2: conditional probabilities of exposure and outcome vertices. Supplemental Figure 3: effect of Epinephrine and SALS intervention assessed by network querying. Supplemental Table 1: definition of terms and detailed criteria used in this study. Supplemental Table 2: effect of epinephrine on prehospital ROSC rate. Supplemental Table 3: multivariable logistic regression model for the effect of SALS protocol on prehospital ROSC and neurologic recovery (6-month CPC score 1 or 2). Supplemental Table 4: effect of SALS intervention on prehospital ROSC and neurologic recovery. [file 8057106.f1.zip › 8057106.f1/4. online_supplements.docx]

ONLINE SUPPLEMENT

Effect of prehospital epinephrine on outcomes of out-of-hospital cardiac arrest: A Bayesian network approach

**Supplemental Tables**

Supplemental Table 1. Definition of terms and detailed criteria used in this study

| Tier | Name of vertex | Description |
| --- | --- | --- |
| Tier 1 | SEX | Biological sex |
|  | AGE | Age (discretized) |
| Tier 2 | CARDIAC | Presumed cardiac etiology |
| Tier 3 | PUBLIC | Cardiac arrest in public location |
|  | WITNESSED | Witnessed cardiac arrest |
|  | RESP.TIME | Response time (discretized time interval between call and arrival of EMTs at scene) |
| Tier 4 | BYST.CPR | Bystander CPR |
| Tier 5 | SHOCKABLE | Shockable initial rhythm |
| Tier 6 | SALS | Application of SALS protocol |
|  | EPI | Use of epinephrine |
|  | ADV.AIRWAY | Advanced airway |
|  | EDA.TIME | Discretized time interval between beginning of CPR by EMTs and ED arrival |
|  | PREH.ROSC | Prehospital ROSC |
| Tier 7 | NEUR.RECOVERY | Neurologic recovery (6-month CPC 1 or 2) |

EMT, emergency medical technician; BLS, basic life support; ED, emergency department; VF, ventricular fibrillation; VT, ventricular tachycardia; AED, automated external defibrillator; ROSC, return of spontaneous circulation

Supplemental Table 2. Effect of epinephrine on prehospital ROSC rate

|  | 0-10 minutes | | 11-20 minutes | | >20 minutes | |
| --- | --- | --- | --- | --- | --- | --- |
| Initial rhythm | HR (95% CI) | *P* | HR (95% CI) | *P* | HR (95% CI) | *P* |
| Non-shockable | 2.02 (1.08-3.78) | 0.028 | 6.94 (4.15-11.61) | <0.001 | 7.43 (2.92-18.91) | <0.001 |
| Shockable | 0.40 (0.21-0.76) | 0.005 | 0.50 (0.32-0.77) | 0.002 | 2.20 (0.76-6.33) | 0.146 |

Supplemental Table 3. Multivariable logistic regression model for the effect of SALS protocol on prehospital ROSC and neurologic recovery (6-month CPC score 1 or 2)

| Prehospital ROSC | | | Neurologic recovery | | |
| --- | --- | --- | --- | --- | --- |
| Predictor | Odds ratio (95% CI) | *p* | Predictor | Odds ratio (95% CI) | *p* |
| SALS | 5.36 (3.54-8.41) | <0.001 | SALS | 1.94 (1.31-2.91) | 0.001 |
| Shockable | 14.79 (9.18-24.36) | <0.001 | Cardiac | 7.81 (3.25-18.08) | <0.001 |
| Cardiac | 7.26 (3.44-14.84) | <0.001 | Shockable | 27.10 (18.60-40.64) | <0.001 |
| SALS:Shockable | 0.38 (0.22-0.66) | 0.001 | SALS:Cardiac | 0.28 (0.11-0.73) | 0.008 |
| SALS:Cardiac | 0.32 (0.15-0.70) | 0.004 |  |  |  |

SALS, smart ALS; ROSC, return of spontaneous circulation; CI, confidence interval

Supplemental Table 4. Effect of SALS intervention on prehospital ROSC and neurologic recovery

| Prehospital ROSC | Non-cardiac | | | | Cardiac | | | |
| --- | --- | --- | --- | --- | --- | --- | --- | --- |
|  | Non-shockable | | Shockable | | Non-shockable | | Shockable | |
|  | OR (95% CI) | *p* | OR (95% CI) | *p* | OR (95% CI) | *p* | OR (95% CI) | *p* |
|  | 5.36 (3.48-8.24) | <0.001 | 2.05 (1.40-3.01) | <0.001 | 1.69 (0.78-3.64) | 0.182 | 0.65 (0.30-1.40) | 0.267 |
| Neurologic recovery | Non-cardiac | | | | Cardiac | | | |
|  | OR (95% CI) | | *p* | | OR (95% CI) | | *p* | |
|  | 1.94 (1.30-2.89) | | 0.001 | | 0.54 (0.23-1.27) | | 0.161 | |

**Legends to supplemental Figures**

**Supplemental fig. 1.** Potential causal pathways allowed to be included in the DAG structure.

**Supplemental fig. 2.** Conditional probabilities of exposure and outcome vertices.

**Supplemental fig. 3.** Effect of Epinephrine and SALS intervention assessed by network querying.
